# Short term cognitive function after sevoflurane anesthesia in patients suspect to obstructive sleep apnea syndrome: an observational study

**DOI:** 10.1186/s12871-021-01363-0

**Published:** 2021-05-18

**Authors:** Soeren Wagner, Lorenz Sutter, Fabian Wagenblast, Andreas Walther, Jan-Henrik Schiff

**Affiliations:** 1 Department of Anesthesiology and Intensive Care, Technical University of Munich, School of Medicine, Klinikum rechts der Isar, Ismaninger Straße 22, 81675 Munich, Germany; 2 Department of Old Age Psychiatry and Psychotherapy, Klinikum Stuttgart, Krankenhaus Bad Cannstatt, Prießnitzweg, 2470374 Stuttgart, Germany; 3 Department of Anesthesiology and Intensive Care, Katharinenhospital Klinikum Stuttgart, Kriegsbergstrasse 60, 70174 Stuttgart, Germany; 4 Philipps-University Marburg Department of Anesthesia and Intensive Care, University Hospital Giessen-Marburg, Marburg Campus, Baldingerstraße, 35033 Marburg, Germany

**Keywords:** Cognitive dysfunction, Sleep apnea, Volatile anesthesia

## Abstract

**Background:**

The obstructive sleep apnea syndrome (OSAS) is characterized by intermittent cerebral hypoxia which can cause cognitive alterations. Likewise, hypoxia induced neurocognitive deficits are detectable after general anesthesia using volatile anesthetics.

The objective of this study was to evaluate the association between a moderate to high risk patients of OSAS and postoperative cognitive dysfunction after volatile anesthesia.

**Methods:**

In this single center prospective, observational study between May 2013 and September 2013, 46 patients aged 55 to 80 years with an estimated hospital stay of at least 3 days undergoing surgery were enrolled. Patients were screened using the STOP-BANG test with score of 3 or higher indicating moderate to high risk of OSAS. The cognitive function was assessed using a neuropsychological assessment battery, including the DemTect test for cognitive impairment among other tests e.g. SKT memory, the day before surgery and within 2 days after extubation.

**Results:**

Twenty-three of the 46 analyzed patients were identified with a moderate to high risk of OSAS. When comparing post- to preoperative phase a significant better performance for the SKT was found for both groups (*p* <  0.001). While the moderate to high risk group scores increased postoperative in the DemTect test, they decreased in the low risk group (*p* <  0.003). When comparing the changes between groups, the moderate to high risk patients showed significant better test result for DemTect testing after anaesthesia. This effect remained robust when adjusting for potential confounding variables using a two-factor ANOVA.

**Conclusion:**

Compared to low risk, a moderate to high risk of OSAS based on the STOP-BANG score was associated with improved postoperative cognitive function measured by the DemTect test.

**Trial registration:**

The study was approved by the local Ethics committee (Ethikkommission der Medizinischen Fakultät der Friedrich-Alexander-Universität Erlangen-Nürnberg, Erlangen, Germany) (reference number: 87_12 B) on 19.04.2012.

## Background

Undergoing surgical procedures, postoperative cognitive dysfunction (POCD) is a frequently occurring complication that impairs several cognitive skills such as attention, speed of intellectual processing memory and executive function [1, 2]. The incidence over different classes of surgery is provided within a range between 8.9 and 46.1% [3]. Though studies tried to define key factors in the development of POCD, its precise pathogenesis and the underlying mechanisms are still unclear [4]. However, the relation of central inflammation and POCD has been approved, while microglia, astrocytes and mast cell dysfunction as well as deficits in neuronal nutrition support contribute to pathological pathways in cognitive decline [5]. Besides age, cognitive reserve, level of education, type of surgery, the anesthetic procedure and reduced or impaired oxygen saturation affects POCD occurrence [6, 7]. Using volatile anesthetic agents such as sevoflurane, clinical studies described its effects on POCD in elderly patients inconsistently and varying conclusions were found. Decreased cognitive function after volatile anesthesia was described [8] as well as advantageous effects after the use of dose dependent sevoflurane anesthesia, which mediate a beneficial cerebral oxygen balance and ameliorate cognitive damage in comparison to none sevoflurane use [9]. However, the latter study reported a sevoflurane-dose dependent improvement of cerebral oxygen supply. Diminished cerebral oxygen saturation seemed to reduce cognitive dysfunction [9]. Likewise, due to respiratory alterations, serious oxygen desaturation and reduced cerebral oxygen supply occur in undetected and undiagnosed obstructive sleep apnea syndrome (OSAS) patients while asleep frequently. The obstructive sleep apnea syndrome is characterized by repetitive complete or partial obstruction of the upper airway track during sleep with preserved effort leading to intermittent hypoxemia [10]. Moreover, it has been reported that OSAS patients usually present with cognitive impairment [11]. So far, attention, episodic memory, working memory and executive function have been identified as the cognitive abilities mostly affected in OSAS patients [12]. In contrast to these findings, during the last few years preconditioning aspects have gained increasing importance. Several studies described that intermitted hypoxia events diminish inflammation, oxidative stress and entail additionally brain tissue protection [13]. Moreover, recently published intermittent hypoxic-hyperoxic training in geriatric patients contributed significantly to improvements in cognitive function [14]. In surgery settings sevoflurane exhibits neuroprotective effects in acute and repeated preconditioning models in particular [15].

These findings raise the question if cognitive dysfunction after surgery using volatile anesthetics could be less pronounced in OSAS patients, whose intermittent nocturnal hypoxic episodes could serve as hypoxic preconditioning. Therefore, we tested the cognitive performance of OSAS suspect subjects before and early after surgery in comparison to matched low risk patients without any OSAS history.

## Methods

In this prospective single-center study two groups (moderate to high risk group and a low risk group) were compared. The study was performed at a tertiary teaching hospital between May 2013 and September 2013 in accordance to the guidelines for Good Clinical Practice and the Declaration of Helsinki for experiments involving humans.

The study was approved by the local Ethics committee (Ethikkommission der Medizinischen Fakultät der Friedrich-Alexander-Universität Erlangen-Nürnberg, Erlangen, Germany) (reference number: 87_12 B, on 19.04.2012). Notable the study approval was given for “general anesthesia” procedures. Due to study conformation a total intravenous anesthesia setting was tested initially [16]. Since surprisingly results and effects has been elicited a complementary study using a volatile anesthesia approach has been established. In the light of basically unchanged study setting under the permission of a “general anesthesia” setting a new ethical vote of the local Ethics committee was dispensable.

### Patients

After written informed consent, 46 adult patients of both sex with an estimated hospital stay of at least 3 days undergoing surgery were enrolled in this study. Inclusion criteria were age between 55 and 80 years and American Society of Anesthesiologists physical status classification of I to III. Patients with a history of brain or head injury, cerebral ischemia, diseases of central nervous system, psychological disorder, alcohol or illicit drug abuse, neuroenhancing or neurocompromising medication, manifest diagnosis of pre-existing cognitive impairment or severe cardiovascular disorder and also patients undergoing cardiac surgery were excluded from the study. Patients were assessed by a detailed screening examination and clinical interview. More patients were screened than were ultimately included in the study, as not every patient screened wanted to take part in the study. Thus, the two study groups were therefore successively replenished. None of the included patients had been treated for OSAS. Cognitive function was assessed using a neuropsychological assessment battery on the day before surgery as a baseline measurement as previously described [16]. The postoperative neuropsychological assessment was performed after surgery on the first or mainly second POD. To avoid a systematic bias and due to the OSAS pathogenesis of one study arm patients have been enrolled in the otorhinolaryngology clinic. Moreover, to achieve a comparable duration of surgery this clinical focused setting seems to be beneficial.

### Assessment of the risk for OSAS

**Fig. 1:**
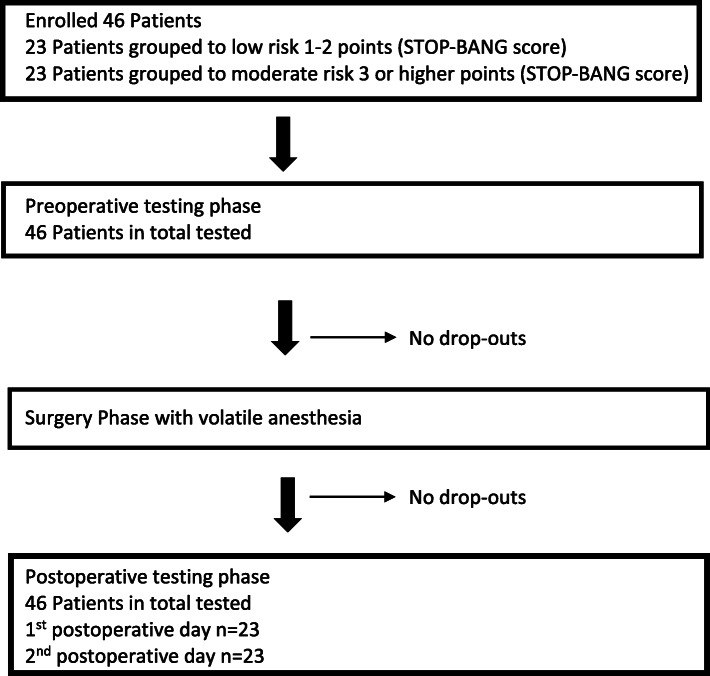
Patients recruitment and follow-up flow chart

The STOP-BANG questionnaire is a brief, international accepted questionnaire for sleep disorders [2]. This validated test detects patients with an increased risk for OSAS reliably [17, 18]. The STOP-BANG questionnaire asks for the incidence of snoring, fatigue and observed stop of breathing, and also considers data on blood pressure, body mass index, age, neck size and gender. Chung and co-workers published the current underlying scoring system for the STOP-BANG questionnaire with an alternative scoring model as an additional assessment/rating approach [19]. Hence, scoring 3 points or higher values patients are classified at least moderate suspect for having an OSA disease. Moreover, male patients scoring values of 3 points are highly suspect for being OSA affected. Thus, if the resulting composite score (range 0 to 8) in our study was 3 or higher, the patient was included into the moderate to high risk group, otherwise the patient was included into the low risk group. If the patients fit the specific study criteria prior surgery, they were asked to participate in the study. After consent the patients were scored via the STOP-BANG questionnaire and grouped as described above (Fig. 1). Notable, the process of patient enrollment and screening has been carried out while the premedication visit of the anesthesiology staff and the patient volume was unpredictable. Moreover, the examiners were blinded to the screened and enrolled participants.

### Clinical protocol

We registered all prescribed medications preoperatively. In particular we ascertained that no neuroenhancing or neurocompromising had been used perioperatively. Benzodiazepines for premedication were strictly avoided in all patients, and instead 75 or 150 μg clonidine was given orally if needed. For anaesthesia induction propofol (propofol 1%; 1.5–2.5 mg/kg bw) and fentanyl (1–5 μg/kg bw) was used. For anesthesia maintenance sevoflurane aimed a MAC of 0.8 to 1.0 and remifentanil (0.2–0.5 μg/kg bw/min) was used. Rocuronium was given as neuromuscular blocking drug. A blood pressure decrease, defined as more than 20% decrease from preoperative untreated, awake systolic blood pressure, were treated immediately. In addition, hypotension, defined as systolic blood pressure values below 80 mmHg, were strictly avoided.

Haemoglobin oxygen saturation was monitored in all patients via pulse oximetry continuously. During the in- and extubation phase, all patients received 100% oxygen for several minutes. Patients were escorted quickly to the recovery room including auxiliary oxygen supply via breathing mask. Additional oxygen was supplied in the recovery room if necessary to raise oxygen saturation and patients were completely weaned of oxygen support before discharge and transfer to a regular ward. Patients´ oxygen saturation before transfer and at arrival at the recovery room is compared statistically. Pain was evaluated by the 11-point numerical rating scale (NRS, 0 = no pain, 10 = maximum pain). Elevated pain scores greater than 4 were addressed by an infusion with 7.5 or 15 mg Piritramide over 20 min according to total body weight.

Focusing on the postoperative phase, low risk patients were monitored in the recovery room continuously for at least 2 h. However, patients in the moderate to high risk group were monitored overnight according to our hospital’s clinical security guidelines on an intermediate care station by telemetry.

### Assessment of cognitive function

Cognitive function was assessed using a neuropsychological assessment battery on the day before surgery as a baseline measurement. Postoperative testing was performed after surgery on the first or second POD. To avoid biases all tests were carried out in a quiet and separate room during daytime and it was attempted to perform pre- and postoperative tests at matching times of the day. The patients’ vision or hearing was not impaired during the course of the study. Existing glasses as visual aids could be worn to the assessments and were not defined as an exclusion criterion. Before the second test in particular, the patient’s well-being was queried so that, for example, discomfort and pain did not falsify the results of the examination. All tests required approximately 60 min per testing phase and patient. In order to avoid learning effects, tests were presented in two different versions if required. Although the Mini-Mental-Status-Test (MMST) is a robust test to estimate the severity and progression of cognitive impairment [4], this test is accused of having learning effects in repeated testing settings. Thus, the assessment battery was adapted and the following tests were performed. Details have been previously described by Wagner and colleagues extensively in 2018 [16]. Briefly:

### DemTect [20]

The DemTect is a highly sensitive psychometric screening test to detect premature mild cognitive impairment and patients with dementia. The cut off values range from 18 to 13 points for adequate age adjusted cognitive performance, 12 to 9 points for mild cognitive impairment and less than 8 points for presumably dementia status.

### Rivermead Behavioural memory test (RBMT): story [21]

The Rivermead Behavioural Memory test is a highly sensitive test to detect impairment of global memory function. The used subtest “story” has a maximum score is 84 points and less values indicates worse performance.

### Zahlen-Verbindungs-test (ZVT) [22]

The ZVT measures cognitive performance speed independent education. While 90 ascending numbers have to be connected with a pen, which are arranged randomly on four different sheets of paper the needed time is recorded. Higher averaged and age-adjusted test values indicate a diminished cognitive performance.

### Trail-making-test (TMT) [23]

The Trail Making Test A/B attempts to test neurocognitive performance combined with psychomotor ability. The time which is needed in the test setting is age adjusted for interpretation. The longer the process takes the worse the cognitive performance is.

### Wechsler memory scale – revised, scale - digital span (digital span) [24]

The Digit Span Test used in our study is a subtest of the Wechsler Memory Scale. The Test analysis the short term memory capacity by using forward and verbal working memory by using backward. Each correct answer leads to a point, which are added in total. Its maximum is 24 points which indicates the best cognitive performance.

### A Short Cognitive Performance Test for Assessing Deficits of memory and Attention (SKT) [25]

The SKT is a short cognitive performance test for detecting memory and attention deficits within a clinical setting. Testing results are expressed in points with a range from zero to 27 points. It is remarkable that a higher test value represents a worse test result and indicates a diminished cognitive performance.

### Color-word-interference-test (FWIT) [26]

The FWIT consists of three parts in which the needed processing time and number of errors are recorded. Higher test values label a worse test performance on analyzed cognitive function of nomination, alertness, and selectivity or rather interference.

### Statistical analysis

For sample size calculation published data of the DemTect have been used, which calculated a standard deviation of approximately 3 points [20]. For clinical relevance we supposed a discrepancy of at least 4 points between the two groups. Thus, we calculated at least 19 needed patients per group to elicit a difference with an α-error of 0.01 and a power of 0.9. The dropout rate for the postoperative testing phase was assumed to be 25%, which lead to a calculated sample size of 25 subjects finally.

The statistical analysis was performed by using IBM SPSS Statistics Version 21.0.0.1 for Microsoft platforms. Metric data were presented as median (range) and categorial data were represented as abundances. Patients with missing data or data influenced by external factors were not included in further analysis. There was no intention to replace the missing or false values. The primary outcome parameter was the change of the cognitive function assessed by the difference between postoperative and preoperative aggregated test values. In addition to the aggregated test values the single scores of the subtest of digit-span and SKT were used to gain a higher differentiation between the groups. The sub scores of “digit-span forward”, “digit-span backward” and of the subgroups defined in the SKT were included in the analysis as a dependent value. For interpretation of the FWIT only the third sequence was considered, because only this sequence can register the executive functions. To calculate the value for the FWIT the processing time of each column was averaged. Outliers were identified using the Grubbs test and were not included in the further data analysis. Deviations from the normal distribution were tested using the Shapiro-Wilk test as a requirement for the secondary two-factor variance-analysis (ANOVA). Differences between baseline values and post-anesthesia values within the two groups were tested for statistical significance using the paired t-test or the Wilcoxon test, respectively. For the primary outcome of the cognitive function differences between the two groups were tested for statistical significance using the unpaired t-test or the Mann-Whitney-U-test, respectively. The distribution of categorial data was tested by the exact test by Fisher. To analyze the secondary outcome parameters for main-effects and interactions with the factor “group” there were used the two-factor variance-analysis. The analyzed variables were: age, sex, nicotine-consumption, alcohol-consumption, duration between extubation and postoperative testing, duration of intraoperative hypertension, lowest perioperative oxygen saturation, presence of a perioperative hypoxia and presence of a severe perioperative hypoxia. Due to the multiple tests, the level of significance was defined as *p* <  0.005 for the two-factor variance-analysis, whereas it was set at *p* <  0.05 for all other statistical tests.

## Results

### Demographics and clinical data

We recruited a total of 46 patients, out of which all patients did complete the second test phase. Overall, no outliers were identified. In Table 1 itemizes the analysis of demographic and related clinical data of the 46 patients. Regarding the postoperative test phase, half of the patients were tested on the first the others on the second POD.

**Table 1:** Patients‘data

	Low risk (***n*** = 23)	Moderate to high risk (***n*** = 23)	***P*** value
**Demographics**			
Age (yrs.)	67.8 (60–84)	68.4 (60.7–76.8)	0.885
Sex (female/male)	12/11	5/18	0.065
Body mass index (BMI) (kg m^−2^)	25.7 (19.4–32.6)	29.4 (23.4–38.1)	0.002*
ASA classification (I/II/III)	4/18/1	0/18/5	0.022*
STOP-BANG score	2 (1–2)	4 (3–6)	< 0.001*
**Comorbidities**			
History of smoking (no/yes/past)	16/6/1	14/2/7	0.041*
Arterial hypertension	6	15	0.017*
Diabetes mellitus (DM)	1	5	0.187
**Surgery**			
Otorhinolaryngologic surgery	19	21	n.s.
Other type of Surgery	4	2	n.s.
**Surgery type details**			
Skin surgery	2	1	1.000
Thyroid gland surgery	2	0	0.489
Ear surgery	8	5	0.514
Nasal and paranasal sinus surgery	4	10	0.108
Salivary gland surgery	7	6	1.000
Midfacial fracture surgery	0	1	1.000
**Operation data**			
Duration of anaesthesia (h)	1.9 (1.1–4.2)	1.8 (1.1–4.3)	0.667
Extubation to 2nd testing time (h)	27.0 (5.9–50.1)	26.4 (19.4–47.5)	0.657
SpO_2_ before procedure (%)	97 (91–100)	97 (89–100)	0.729
SpO_2_ at before transfer to intermediate care (%)	99 (87–100)	98 (92–100)	0.819
SpO_2_ at arrival intermediate care (%)	95 (86–100)	92 (88–96)	0.073
SpO_2_ lowest level (%)	93 (85–98)	91 (81–96)	0.141
**Postoperative testing day**			
First postoperative day	1	22	n.s.
Second postoperative day	0	23	n.s.

Data are reported as number or as median (range). SpO_2_ is the oxygen saturation measured by pulse oximetry. *P* value is the significance level of the difference between the two groups. (*n.s.* not significant)

The patients allocated to the moderate to high risk group had a higher score in the STOP-BANG test and therefore a higher risk for OSAS as compared with the patients in the low risk group. The average age was somewhat higher in the moderate to high risk group, while the percentage of males and the BMI was significantly higher in the moderate to high risk group than in the low risk group. Obesity and male sex are risk factors for sleep apnea, both parameters are used to identify patients with a high susceptibility to suffering from OSAS in the STOP-BANG questionnaire. The body mass index (BMI) was significantly higher in the moderate to high risk group. Analyzing further questionnaire items, the moderate to high risk group showed a significantly higher proportion of arterial hypertension, DM and medication with betablocker (*p* = 0.047) or ACE/AT1 inhibitors (*p* = 0.047), with higher ASA physical status. Nicotine use was found in the moderate to high risk group significantly more frequent than in the low risk group (*p* = 0.041). The main type of surgery was otorhinolaryngologic surgery and no significant differences regarding dosage and anesthesia procedure between the moderate to high risk and the low risk group were noticed (Table 1).

Although all patients received supplementary oxygen during transfer to the recovery room, we observed in both groups a decrease in oximetry parameters. However, a clearly more pronounced desaturation in the moderate to high risk subjects after transfer to the recovery room was found (Table 2). We did not find a significant difference between both groups regarding the lowest and the highest oxygen saturation. Within the same period of time moderate to high risk patients decreased more pronounced in comparison to the low risk group while approaching to the recovering room (*p* = 0.073). Furthermore, between both groups no significant difference regarding serious hypoxic episodes was detected (*p* = 0.722). Serious hypoxic episodes with a duration of ≥1 min and decrease to values lower than 90% were detected in 6 low risk patients and in 4 moderate to high risk patients. In both groups we found postoperative hypoxic episodes in 17 low risk patients and in 22 moderate to high risk patients. The overall decrease of oxygen saturation was more accentuated in the moderate to high risk than in the low risk group.

**Table 2:** Oximetry data before and after transfer to the recovery room

	SpO_2_ at before transfer to recovery room (%)	SpO_2_ at arrival recovery room (%)	*P* value
**Low risk (*n*** **= 23)**	99 (87–100)	95 (86–100)	0,021
**Moderate to high risk (*n*** **= 23)**	98 (92–100)	92 (88–96)	< 0,001

Data are reported as number or as median (range). SpO_2_ is the oxygen saturation measured by pulse oximetry. *P* value is the significance level of the difference between the two groups

#### Neuropsychological assessment

A combination of neuropsychological assessments determines the patient’s cognitive function on the day before surgery as a baseline measurement. The same test battery was used on the first or second POD, though tailored to suit the B version of certain test was presented. The pre- and postoperative test results are summarized in Tables 3 and 4. The primary analysis quantifies the differences between the two groups by comparing the differences of the pre- and the postoperative cognitive test values as the primary outcome of this study (Table 5). Here we found a significant difference in only the DemTect Test between the two groups (*p* = 0.026). While the DemTect result decreased in the low risk group, the result in the moderate to high risk group was unchanged. This statistical significance was confirmed by the secondary analysis ANOVA which revealed high significance for the factor “group” but no significance for the factor “sex” and the interaction between the two factors.

**Table 3:** Results of the pre- and postoperative neurocognitive testing within each group

Test	Low risk group			Moderate to high risk group		
	Pre	Post	***P*** value	Pre	post	***P*** value
DemTect (points)	16 (12–18)	14 (10–18)	< 0.003*	17 (13–18)	18 (11–18)	1.0
RBMT (points)	35 (13–52)	32 (18–66)	0.455	38 (19–56)	42 (16–58)	0.079
ZVT (min)	1.7 (1.2–2.8)	1.5 (1.0–2.1)	< 0.001*	1.7 (1.0–2.3)	1.6 (1.0–2.3)	0.001*
TMT (min)	1.4 (0.9–3.0)	1.3 (0.7–2.1)	0.054	1.5 (0.9–2.7)	1.3 (1.0–3.3)	0.174
Digit Span Forwards (points)	8 (5–11)	8 (4–12)	0.925	8 (5–12)	8 (6–12)	0.498
Digit Span Backwards (points)	6 (3–10)	7 (5–10)	0.003*	6 (3–12)	6 (3–12)	0.245
Digit Span Total (points)	14 (8–21)	15 (10–21)	0.057	14 (10–24)	15 (9–24)	0.617
FWIT (sec)	26.1 (19.1–66.1)	25.7 (19.4–47.1)	0.223	30.2 (16.5–50.2)	30.1 (15.4–45.0)	0.192
SKT Attention (points)	0 (0–1)	2 (0–5)	< 0.001*	0 (0–2)	1 (0–4)	< 0.001*
SKT Memory (points)	1 (0–3)	1 (0–5)	0.817	1 (0–6)	1 (0–4)	0.074
SKT Total (points)	1 (0–4)	3 (1–7)	< 0.001*	1 (0–6)	3 (0–6)	< 0.001*

Data are reported as median (range). The p value is the significance level of the difference between pre- and postoperative values within a group

**Table 4:** Results of the pre- and postoperative neurocognitive testing between each group

Test	Pre operative			Post operative		
	Pre low risk	Pre moderate to high risk	***P***_ANOVA_ value	Post low risk	Post moderate to high risk	***P***_ANOVA_ value
DemTect (points)	16 (12–18)	17 (13–18)	0.561	14 (10–18)	18 (11–18)	0.013*
RBMT (points)	35 (13–52)	38 (19–56)	0.292	32 (18–66)	42 (16–58)	0.157
ZVT (min)	1.7 (1.2–2.8)	1.7 (1.0–2.3)	0.780	1.5 (1.0–2.1)	1.6 (1.0–2.3)	0.864
TMT (min)	1.4 (0.9–3.0)	1.5 (0.9–2.7)	0.958	1.3 (0.7–2.1)	1.3 (1.0–3.3)	0.534
Digit Span Forwards (points)	8 (5–11)	8 (5–12)	0.804	8 (4–12)	8 (6–12)	0.804
Digit Span Backwards (points)	6 (3–10)	6 (3–12)	0.760	7 (5–10)	6 (3–12)	0.557
Digit Span Total (points)	14 (8–21)	14 (10–24)	0.669	15 (10–21)	15 (9–24)	0.827
FWIT (sec)	26.1 (19.1–66.1)	30.2 (16.5–50.2)	0.211	25.7 (19.4–47.1)	30.1 (15.4–45.0)	0.219
SKT Attention (points)	0 (0–1)	0 (0–2)	0.760	2 (0–5)	1 (0–4)	0.207
SKT Memory (points)	1 (0–3)	1 (0–6)	0.229	1 (0–5)	1 (0–4)	0.778
SKT Total (points)	1 (0–4)	1 (0–6)	0.494	3 (1–7)	3 (0–6)	0.653

Data are reported as median (range). The p _ANOVA_ value is the significance level of the difference between pre- and postoperative values within a group; *: significant differences with *p* < 0.05

**Table 5:** Change of cognitive functions, expressed as the difference between pre- and postoperative values

Test	Low risk	Moderate to high risk	P_**MWU**_	P_**ANOVA**_
DemTect (points)	-2 (−6–3)	0 (−4–5)	0.026*	0.009*
RBMT (points)	0 (−9–15)	4 (− 16–20)	0.443	0.993
ZVT (min)	−11 (− 25–7)	− 7 (− 25–11)	0.397	0.220
TMT (min)	−15 (− 52–34)	− 11 (− 41–60)	0.507	0.319
Digit Span Forwards (points)	0 (− 3–3)	0 (− 2–2)	0.573	0.448
Digit Span Backwards (points)	1 (−1–3)	0 (− 2–3)	0.162	0.102
Digit Span Total (points)	1 (− 1–6)	0 (− 2–4)	0.229	0.106
FWIT (min)	−4 (− 52–64)	− 5 (− 21–29)	0.744	0.769
SKT Attention (points)	2 (0–4)	1 (0–3)	0.080	0.011*
SKT Memory (points)	0 (−1–4)	0 (− 2–2)	0.205	0.307
SKT Total (points)	2 (− 1–6)	1 (− 1–4)	0.611	0.008*

Data are reported as median (range). P_MWU_ is the significance level of the difference between the two groups obtained by the Mann-Whitney test. P_ANOVA_ are the significance levels of the interaction between the factors listed above and “gender” with the “group” as cofactor obtained by analysis of variance

The other factors such as age, nicotine-, alcohol-consumption, duration between extubation and postoperative testing, etc. tested with ANOVA revealed statistically no significant result.

The preoperative test values between both groups did not differ significantly, whereas, postoperatively, significant differences between both groups were found for the DemTect, where the patients in the low risk group showed significantly worse results in comparison to the moderate to high risk group (*p* = 0.013). The other comparison revealed statistically no significant result. When comparing pre- and postoperative values within the low risk group, there was a significant decrease of test results in two of the tests systems (DemTect (*p* = 0.003), SKT (*p* = 0.001), see Table 3). In contrast, the patients in the moderate to high risk group showed a significant decrease in the SKT test system only (*p* = 0.001) as well as a tendency increase in the DemTect test (n.s.). Both groups enhanced their test results regarding the ZVT system significantly (*p* = 0.001). (Tables 3,4, 5).

## Discussion

The aim of this study was to test for possible beneficial associations from intrinsic hypoxemic episodes in patients with a moderate to high risk for OSAS on early postoperative cognitive deficits after non-cardiac surgery using volatile anesthesia. When comparing the two study groups, we discovered a significant difference between both groups in the DemTect test regarding the preoperative to postoperative testing phase in favor of the moderate to high risk of OSAS group.

With respect to education level and possible interindividual variation in testing values we calculated the change in test results prior to after surgery for each patient separately and consecutive for each group. We found an isolated significant change between moderate to high risk and low risk group patients in respect to the pre- and postoperative period in the DemTect Test. Surprisingly, the low risk group showed a significant decrease of test performance, in the moderate to high risk group a tendency to increased values was observed. Both groups demonstrated concordant, significantly increasing test results for the ZVT and decreasing results in SKT scores attention test section. Test values for digit span backwards testing in the low risk group demonstrated significant better results in the early post-operative testing phase.

The non-difference between groups with exception of the DemTect need to be explained. Briefly, executive function is a multidimensional cognitive process which includes skills like behavioral inhibition, process planning, mental flexibility, working memory and motor function. One possible explanation is the difference in the test construction. The DemTect assesses different cognitive domains in terms of attention, memory and executive function, whereas the other tests focus only on one or two domains. Therefore, subjects must elicit a more pronounced decrease in a single cognitive domain to receive a noticeable result than in multiple cognitive domains to achieve a significant test result. Another is a higher sensitivity and specificity of the DemTect in comparison to the SKT. Hahn and Kessler demonstrated that even less marked cognitive dysfunction could be detected via DemTect test with high sensitivity and specificity [27].

While the DemTect detected impairments in executive function for the low risk group, the FWIT, which also tests for deficits in executive function, could not find any significant results. Working memory was tested in particular using the Digit Span test, psychomotor speed was analyzed via TMT and ZVT to the same results in both groups with a tendency of increasing speed.

It is commonly accepted that moderate to high risk patients exhibit limited cognitive ability and deficits in the quality of psychomotor speed and executive function, while memory functions, motor control, construction, attention, and speed of processing abilities were less influenced [28]. However, other studies comparing moderate to high vs. low risk patients failed to detect any differences in e.g. executive function [29, 30]. Other cognitive functions were found inconsistent or inconclusive in previous published studies. In accordance we could not detect any significant preoperative differences in cognitive performance in this study. There remains controversy about the cognitive performance of moderate to high risk patients and the grade of cognitive impairment. Moreover, a recently published work using the same assessment battery demonstrated diminished performance values focusing on several cognitive qualities [16]. This might be explained by the fact that the detected STOP-BANG Score for moderate to high risk suspect patients averaged 4 in comparison to a score of 5 in the cited study. Recently, STOP-BANG Score of 5 in screened patients attributed a high risk for OASA while patients showing a score of 4 had a mixed risk-factor between midrange and high risk [19]. Taking these variations into account the present work reflects an overall high probability for the moderate to high risk group compared with low risk patients. Therefore, other reasons must be responsible to explain our current results.

Our findings might be explained by the concept of cognitive reserve, which could compensate or disguise the postoperative decrease in cognitive function [31]. Following this concept, the moderate to high risk group might not show a decrease in cognitive performance even if the DemTect test reveals small changes in cognitive function. However, postoperative decline in cognitive function following anesthesia is a well-known but poorly understood phenomenon.

We found a significant improvement in ZVT and significant impairment in SKT scores. Moreover, the moderate to high risk group gained statistically the same values as the low risk group. Surprisingly, we found inversely calculated results regarding the DemTect test. While low risk patients elicited a significant decrease in test results the moderate to high risk patients demonstrated an increased in their test score. This inconclusive finding leads to the assumption that there might be some other reason beside cognitive reserve and test quality aspects.

Alongside patient associated causes, anesthesia related factors on cognition are discussed in literature. Additional hypoxic preconditioning aspects might play a role. Obstructive apnea patients show severe oxygen desaturation while asleep. During these phases’ patients experience hypercarboxic hypoxemia. Hypoxemic and ischemic preconditioning benefits have been discussed in literature for 30 years [32, 33]. Recent research using remote ischemic conditioning technic demonstrate encouraging data regarding organ protection particularly in cardiac and brain tissue [34–36]. On the other hand, since the ISPOCD1 study it is believed that no correlation between a perioperative hypoxic phase and POCD exists [37]. The authors focused on pulse oximetry data with a desaturation to more than 80% for a minimum period of 2 min. Only in 11% of their included patients fit to these criteria. Using a less severe definition for hypoxia, more cases of hypoxia were found within our observed study group. Thus 79% of low risk patients and 96% of moderate to high risk patients presented a hypoxic episode if the critical value is considered to be 95% oxygen saturation. Although this phenomenon is unwanted, it appears regularly in the perioperative setting and is a well-known complication after the operation process [38, 39]. These hypoxic episodes can persist for more than 16 h and have been described within the second and third night after operation as well [40].

Remote ischemic preconditioning seems to have a positive impact on cognitive function of elderly patients [41]. In humans, remote ischemic preconditioning effects still remains conflicting. Nevertheless, in 2016 a publication reported a promising beneficial effects of hypoxic training in older people particular focusing cognitive function [42]. These findings were confirmed in 2017 [14]. Moreover, these results are notable regarding the used test system DemTect utilized in our study. Bayer and colleagues report a significant improvement in DemTect test and Clock Drawing Test values in the treated group while the low risk group failed to improve their test results.

### Limitations

There are some study limitations that should be paid attention to. The Gold Standard to classify the severity of OSAS is the polysomnography using a sleep laboratory facility. The apnea/hypopnea index (AHI) resulting from measured respiratory disturbances characterized the different level severity. Dramatic oxygen desaturation far below the physiological range indicates as a key clinical parameter an apnea episode during sleep. However, this method is laborious, cost-intensive and time-consuming. Therefore, several validated screening tools as cost-efficient alternatives are established und used worldwide to screen for OSA quickly. Thus, we used the STOP-BANG questionnaire to identify patients with a moderate to high risk for OSAS in the current study. The used STOP-BANG questionnaire screened the risk for an OSAS which potentially leads to apnea phases while sleep. This consequently means that the screened patients have not been diagnosed for OSAS using a sleep laboratory tests or polysomnographic studies. Additionally, the STOP-BANG test is an international well-established screening tool to quantify the risk for having the OSAS. While a STOP-BANG score of 5–8 identifies patients with a high probability of moderate or severe OSAS [43]. A score of 3 or higher has been confirmed to offer a high sensitivity to identify sleep apnea and seems to be a suitable cutoff value for screening purpose [19, 44], as even with a mild status of OSAS oxygen desaturation occurs during sleep [45]. Furthermore, we supposed that the patients in our moderate to high risk group were at least suspected of being affected to intermittent hypoxemic episodes while sleep.

Although we used patients with the same surgical procedures and matching accompanied characteristic regarding some typical OSAS issues the groups are different. A two-way ANOVA with sex and group as factors revealed that the observed differences in the change of the cognitive function could be attributed to the factor group whereas sex did not show a significant effect. Additionally, we did not perform alpha adjustment for multiple testing, however this finding was confirmed by secondary analyses including ANOVA. Therefore, we think that the chance for a type I error with regard to the isolated finding in the DemTect test is low.

Moreover, the study focused on non-cardiac surgery patients only. As O’Brien and colleagues summarized that intraoperative hypotension is an intrinsic risk factor of postoperative cognitive dysfunction [46], intraoperative hemodynamic stability was considered carefully in this study. We did not observe hypotension, defined as systolic blood pressure values below 80 mmHg [47]. Short-term hypotension phases between the measurement intervals might remained undetected and even minor periods of hypotension are clearly associated with worse cognitive function in the postoperative phase. Additionally, the wide range in subjects’ intellectual function and education might went unrecognized and impacted on our results. Furthermore, we did not analyze any inflammatory marker in our study and did not measure the exact depth of anesthesia. A MAC of 0.8–1.0 of volatile anesthesia is known as a well-established anesthesia.

Finally, this study included only volatile anesthesia to avoid additional confounding factors. Furthermore, no multivariant analysis for confounder control was made due to the relatively small sample size under observation. In future studies, we will need to evaluate the impact of hypoxic preconditioning on post-operative cognitive function and further elicit which anesthetic approach is suitable and feasible for which type of patient to avoid cognitive decline after surgery.

In the present study, we aimed to evaluate the influence of repeated hypoxic phases while asleep in patients with a high probability for moderate to high risk group. We used the STOP-BANG questionnaire to detect suspect patients and analyze their cognitive function after surgery with volatile anesthetics. We used an assessment battery to evaluate cognitive function and found that the scores in the moderate to high risk group were almost the same as in the low risk group. With exception in the DemTect test which showed a significant better result for the moderate to high risk group in the postoperative testing phase in comparison to the low risk group. The post-surgery scores in the moderate to high risk group were mainly the same as preoperatively regarding the other used tests. Likewise, these results have been found in the low risk group. Nevertheless, largely the differences still remain insignificant.

## Conclusions

In conclusion, an increased risk of OSAS based on the STOP-BANG score is associated with a reduced postoperative decline in DemTect performance.

Based on these findings, future studies should investigate whether chronic, repetitive hypoxemic episodes that are typically observed in patients with increased risk of OSAS may have a beneficial influence on postoperative cognitive function decline and should provide insight into potential advantageous effects of different types of anesthesia.

## Data Availability

The datasets generated and analyzed during the current study are available from the corresponding author on reasonable request.

## Abbreviations

OSAS: Obstructive Sleep Apnea Syndrome
ASA: American Society of Anesthesiologists physical status classification
AHI: Apnea/Hypopnea Index
STOP-BANG: Snoring, Tired, Observed, Pressure, Body mass index, Age, Neck size, Gender
TCI: Target Controlled Infusion
NRS: Numerical Rating Scale
RBMT: Rivermead Behavioral Memory Test
ZVT: Zahlen-Verbindungs-Test
TMT: Trail-Making-Test
SKT: Short Cognitive Performance Test
FWIT: Color-Word-Interference-Test
SASM: Society of Anesthesia and Sleep Medicine
BW: Body weight
DM: Diabetes mellitus
BMI: Body mass index
MAC: Minimal Alveolar Concentration
